# Nonrigid registration of medical image based on adaptive local structure tensor and normalized mutual information

**DOI:** 10.1002/acm2.12612

**Published:** 2019-05-23

**Authors:** Tiejun Yang, Qi Tang, Lei Li, Jikun Song, Chunhua Zhu, Lu Tang

**Affiliations:** ^1^ College of Informational Science and Engineering Henan University of Technology High‐Tech Zone Zhengzhou City China

**Keywords:** local structure tensor, multimodality image, nonrigid registration, normalized mutual information, spatial information

## Abstract

Nonrigid registration of medical images is especially critical in clinical treatment. Mutual information is a popular similarity measure for medical image registration; however, only the intensity statistical characteristics of the global consistency of image are considered in MI, and the spatial information is ignored. In this paper, a novel intensity‐based similarity measure combining normalized mutual information with spatial information for nonrigid medical image registration is proposed. The different parameters of Gaussian filtering are defined according to the regional variance, the adaptive Gaussian filtering is introduced into the local structure tensor. Then, the obtained adaptive local structure tensor is used to extract the spatial information and define the weighting function. Finally, normalized mutual information is distributed to each pixel, and the discrete normalized mutual information is multiplied with a weighting term to obtain a new measure. The novel measure fully considers the spatial information of the image neighborhood, gives the location of the strong spatial information a larger weight, and the registration of the strong gradient regions has a priority over the small gradient regions. The simulated brain image with single‐modality and multimodality are used for registration validation experiments. The results show that the new similarity measure improves the registration accuracy and robustness compared with the classical registration algorithm, reduces the risk of falling into local extremes during the registration process.

## 1 INTRODUCTION

Image registration refers to the process of finding a spatial transformation to make corresponding points of different images reach the same spatial position and anatomical position; these images are obtained by different equipment, different time, or different environments. With the rapid development of modern medical imaging technology, images with various types of information have emerged, such as computed tomography (CT), magnetic resonance imaging (MRI), positron emission tomography (PET), etc. CT has a clear image to the bone structure and can accurately locate the lesion, but the image effect on the soft tissue is not good. MRI has significant advantages in reflecting soft tissue information, but is not sensitive to calcified regions and subject to geometric distortion due to magnetic interference. PET can clearly observe the metabolism of various organs, but due to the low pixel resolution, the structure and regional contour of the organs cannot be clearly found. Due to the different imaging principles of various medical devices, the medical image information is greatly different. The single‐modality image provides unilateral information for the doctor’s clinical diagnosis, but in order to obtain more complete and complementary image information, it is necessary to fuse various types of information so that doctors can make more accurate and reliable diagnosis.

Currently, the similarity measures of nonrigid medical image registration are mainly divided into feature‐based method and intensity‐based method^1^; the former usually uses some points, lines, area, and edges of the image as feature information, the registration method has advantages of small computational cost and high efficiency, but is not easy to calculate automatically, the image registration effect is also easily affected by factors such as the level of the operator and the accuracy of feature point selection. The intensity‐based method directly uses the intensity information of image, which avoids the error in image registration caused by the feature extraction process. For simplicity, intensity‐based similarity measures are typically used instead of feature‐based similarity measures. In the field of intensity‐based method, similarity measures can be divided into two categories: one method directly uses the intensity of the image pixel which contains the sum of squared distances^2^ (SSD) and the gradient difference^3^ (GD). Another method which based on information theory mainly considers the intensity distribution of pixel points and uses statistical entropy strategy. Mutual information^4^ (MI) and normalized mutual information^5^ (NMI) belong to this category. Among them, the method based on the mutual information is the most widely used, but it only considers the intensity distribution of image, ignoring the spatial and geometric information of image completely.

Pluim et al.^6^ proposed a similarity measure called gradient mutual information (GMI) by combining MI with gradient, it improved robustness of registration. It is not an extended measure of MI, but just the addition of the multiplicative factor todescribe the neighborhood information. Russakoff^7^ proposed the regional mutual information (RMI), which calculates the MI of two images in the local range, can reflect the intensity distribution of local image and obtain better robustness than traditional MI similarity measures. However, it needs to calculate the entropy of probability distribution with higher dimensional. Loeckx^8^ proposed the conditional mutual information similarity measures, which uses spatial information as an additional channel to calculate the joint probability distribution. The algorithm improves the registration accuracy, but the corresponding anatomical structures are further spatially separated, some structural features are ignored. Rivaz^9^ proposed α‐mutual information called self‐similarity α‐MI (SeSaMI), which makes full use of the local feature structure of the image to enhance the robustness of MI. Luan^10^ proposed the qualitative measure of mutual information, which adds the utility coefficient to the traditional calculation of MI, the calculation process is complicated and difficult to practical application. Hossny^11^ proposed a local structural mutual information registration method which divides the image space into multiple spaces to estimate MI independently, and uses the weighted sum of MI as the similarity measure, local structural similarity index is preserved. Wang Jun^12^ proposed a B‐spline and regional mutual information (BRMI) registration method. The image is regarded as the distribution of multidimensional points, each point represents a pixel and its neighboring point pixels, by calculating the information entropy of multidimensional points, the local mutual information of the two images is obtained. The method effectively improves the accuracy of registration but the efficiency is reduced.

Qu Jiahui^13^ proposed a hyperspectral image fusion algorithm using structure tensor, which introduces structure tensor to extract the spatial details of enhanced PAN images. Experiments show that the spatial information extracted by the horizontal gradient and vertical gradient only retains the edge information of the original image, but the spatial information obtained by the structural tensor method contains a large number of edges and structural information. James M. Sloan^14^ describes a novel structural image descriptor for image registration called the fractionally anisotropic structural tensor representation (FASTR). It does not depend on voxel intensities absolutely, and is insensitive to the image which has a slowly varying intensity inhomogeneity. The results show that FASTR would produce more accurate results than MI towards the images with distinct intensity inhomogeneity. However, the proposed similarity measure can only be used for rigid medical image registration with simple deformations such as translation, rotation or scaling. In fact, because medical images have local correspondence missing and complex nonlinear deformations, as well as the irregular physiological movements of the organs, nonrigid registration is necessary and can fully describe the spatial relationship between images. In this paper, a new similarity measure based on adaptive local structure tensor and NMI is proposed, in which spatial information, geometric information and mutual information are combined to improve the similarity measure. The nonrigid registration with large deformation is considered.

The contributions of this paper have following four aspects:

1. In order to reflect the local structure information, adaptive Gaussian filtering is introduced into the local structure tensor, and the parameters of Gaussian kernel function are defined according to the regional variance of image, which can better protect the image details.
2. Discrete NMI is defined according to the contribution of pixel points to the total similarity measure, which is beneficial to the combination with spatial information.
3. The spatial information extracted from the adaptive local structure tensor is used to customize the weighting function. It multiplies with the discrete NMI to obtain a new measure function called adaptive local structure tensor‐normalized mutual information (ALST‐NMI).
4. The algorithm in this paper is used to register the brain images with single‐modality and multimodality, the accuracy and effectiveness of registration are both improved.

## 2 METHODS

### 2.1 The registration framework

Medical image registration mainly includes four modules: transformation model, similarity measure, optimization algorithm, and interpolation algorithm. First, the appropriate transformation model is selected according to the specific application, and determines the spatial transformation method of the floating image. Second, a similarity measure is defined to measure the degree of similarity between the reference image and floating image after transformation. In this way, it is judged whether two images have been correctly matched. Third, an interpolation algorithm is used to assign the intensity value to pixels in the image, and the current similarity measure is obtained by comparison with the reference image to determine whether the next round of optimization is needed to update the parameters. Finally, a specific optimization algorithm is used to search for the best transformation result continuously until the similarity of the two images is maximized.

**Figure 1:**
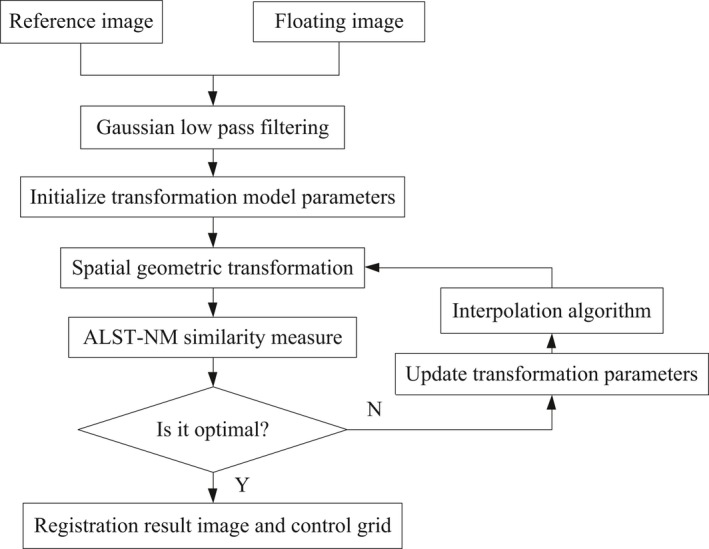
Diagram of the proposed registration algorithm.

The flow diagram is shown in Fig. 1. In this paper, B‐spline is selected as the transformation model, ALST‐NMI is the similarity measure, and the steepest gradient descent method is used as the optimization algorithm for the registration experiment.

### 2.2 Registration model and optimization solution strategy

#### 2.2.1 B‐spline transformation model

The nonrigid registration method based on B‐spline has been widely used by scholars because of its universality, smoothness, and computational efficiency. First, we perform mesh refinement on the registration image, then the pixels become control points. Second, the displacement of each control point in each direction is solved by the optimization algorithm, so any nonlinear transformation on the image can be simulated. Finally, the movement of each control point forms a control grid with nonlinear deformation, the control grid is applied to the floating image to obtain the B‐spline transformed registration image.

In this paper, the cubic B‐spline is selected as the transformation model. The two‐dimensional (2D) cubic B‐spline transformation can be expressed as follows:

(1) $$T\left(x,y\right)=\sum_{m=0}^{3}\sum_{n=0}^{3}B_{m}\left(u\right)B_{n}\left(v\right){\mathrm{phi}}_{i+m,j+n}$$

where Φ represents the control point $i=\left\lfloor \frac{x}{P_{x}}\right\rfloor -1,j=\left\lfloor \frac{y}{P_{y}}\right\rfloor -1,u=\frac{x}{\mathrm{Px}}-\left\lfloor \frac{x}{P_{x}}\right\rfloor ,v=\frac{y}{\mathrm{Py}}-\left\lfloor \frac{y}{P_{y}}\right\rfloor$, $\left\lfloor \cdot \right\rfloor$ represents the next rounding, and B_m_(u) represents the m‐th cubic B‐spline basis function.

(2) $$\left\{\begin{array}{c} B_{0}\left(u\right)=\frac{{\left(1-u\right)}^{3}}{6}\\ B_{1}\left(u\right)=\frac{\left(3u^{3}-6u^{2}+4\right)}{6}\\ B_{2}\left(u\right)=\frac{\left(-3u^{3}+3u^{2}+3u+1\right)}{6}\\ B_{3}\left(u\right)=\frac{{\left(u\right)}^{3}}{6} \end{array}\right.$$

#### 2.2.2 Optimization algorithm

The purpose of image registration is to find a spatial transformation to make corresponding points of the different images reach the same spatial position and anatomical position. The registration problem is transformed into the optimal solution of the cost function, then the optimal transformation parameters are found to minimize the cost function.

(3) $$\mu =\arg\;\min_{\mu }\Phi \left(f_{R},f_{F}\circ g\left(∙|\mu \right)\right)$$

where the cost Φ function is a negative ALST‐NMI similarity measure function, *g* is a nonrigid transformation function, and the symbol ∘ represents a compound operation of *f_F_* and function *g*.

The optimization strategy refers to the search optimization process in which the spatial transformation parameters are continuously adjusted in the image registration, so that the similarity measure is maximized and the images are aligned as much as possible. An applicable optimization strategy can not only improve the computational efficiency of the algorithm, but also obtain more accurate optimization results. Therefore, the steepest gradient descent method is used to iterative update the parameter value along the direction of the gradient descent, as shown in the eq. (4).

(4) $$\mu_{k+1}=\mu_{k}-a_{k}\nabla \Phi \left(\mu_{k}\right)$$

where *a_k_* is the search step size and ∇Φ(μ_k_) is the gradient of the cost function.

### 2.3 ALST‐NMI similarity measure

Normalized mutual information measure is a commonly used similarity measure, which can accurately represent the similarity among intensity images. It can also effectively solves the problem that overlap region in the image affects the registration accuracy in MI registration process, and ensure the effectiveness. However, the registration algorithm based on the NMI does not consider the spatial information of the image, resulting in low accuracy even misleading registration. Gradient can effectively describe local structure information and is used to estimate local geometry information of image widely^15^. However, the gradient is sensitive to noise, and the positive and negative gradients are similar on both sides of the edge in the image, which will causes the gradient information to counteract in the smoothing algorithm. The local structure tensor does not produce the problem abovementioned, and can also still be extracted under the condition of the local gradient loss. At the same time, it^16^, ^17^, ^18^ directly uses the image intensity matrix to perform operations, which can effectively preserve the structural and gradient information of the image pixel, provide a more meaningful description than the gradient information. Therefore, this paper proposes a new similarity measure which combines NMI and local structure tensor.

#### 2.3.1 Discrete normalized mutual information

The NMI can be expressed as:

(5) $$\text{NMI}=\frac{H(R)+H(F)}{H(R,F)}$$

where R and F represent the reference image and floating image namely, H(R) and H(F) denote the information entropy of R and F, and H(R,F) is the joint information entropy of two images.

Normalized mutual information is distributed to each pixel, discrete NMI can be defined by the contribution of pixel points to the total similarity measure:

(6) $$\text{NMI}\left(x,f_{R},f_{F}\right)=\frac{\frac{1}{N_{1}}\log_{2}\left(P_{R}\left(x\right)\right)+\frac{1}{N_{2}}\log_{2}\left(P_{F}\left(x\right)\right)}{H(R,F)}$$

where N_1_ and N_2_ is the number of pixels accumulatively used in the reference image and floating image, x represents the position of pixel in *f_R_* and *f_F_*, and P_R_(x) and P_F_(x) are the marginal probabilities of the x.

#### 2.3.2 Adaptive local structure tensor

The local structure tensor of each point is shown in eq. (7):

(7) $$LST\left(i,j\right)=G_{\sigma }*\left(\nabla I*\nabla I{\left(i,j\right)}^{T}\right)=\left[\begin{array}{c} I_{x}^{2}*G_{\sigma }\;\;\;\;\;I_{x}I_{y}*G_{\sigma }\\ I_{x}I_{y}*G_{\sigma }\;\;\;\;I_{y}^{2}*G_{\sigma } \end{array}\right]$$

where $G_{\sigma }={\left(\sigma \sqrt{2\pi }\right)}^{-1}\exp\left(-{\left|x\right|}^{2}/2\sigma^{2}\right)$ is a 2D Gaussian kernel function, σ^2^ represents the variance of Gaussian kernel function, * represents a convolution operation, ∇ represents the gradient operation, and the superscript T indicates the transpose of the matrix.

The traditional local structure tensor method selects fixed filter parameters for entire image, but the size of Gaussian kernel function variance has a great influence on the weight of the Gaussian template. The smaller variance of Gaussian kernel function is, the smaller the weight of the noncentral pixel is. Thus, the role of the neighborhood in the filtering process is almost ignored, and it degenerates into the point operation of the image, which does not achieve the effect of denoising. When the variance of Gaussian kernel function is larger, the Gaussian filtering degenerates into a mean template, which is likely to cause loss of image detail. Therefore, only the appropriate value of Gaussian kernel function variance is selected, the details of the image can be retained, and noise can be reduced.

In order to protect the image details and denoise, the adaptive Gaussian filtering is introduced into local structure tensor. First, the local structure is judged as a consistent region or a region based on edge and corner. If it belongs to the region based on edge and corner, the degree of dispersion in the region is relatively large, the variance of the corresponding pixel value tends to be large; if it belongs to the consistent region, the degree of dispersion in the region is relatively small, and the variance of the corresponding pixel value is small. Therefore, we calculate the regional variance, first, then the variance of Gaussian kernel function and the Gaussian template are adaptively selected according to the regional variance. The larger the regional variance is, the Gaussian kernel function with smaller variance and smaller Gaussian template is selected; the smaller the regional variance is, the Gaussian kernel function with larger variance and larger Gaussian template is selected.

The formula for regional variance is:

(8) $$D=\frac{\sum_{(i,j)\in S}{\left(x_{\mathrm{ij}}-\bar{x}\right)}^{2}}{i\cdot j},\text{while}\;\;\bar{x}=\frac{\sum_{(i,j)\in S}x_{\mathrm{ij}}}{i\cdot j}$$

where D is the regional variance and *x_ij_* represents the point in the S region

#### 2.3.3 ALST‐NMI similarity measure

The singular value decomposition is performed on local structure tensor to obtain non‐negative eigenvalues which is denoted as λ_1_, λ_2 _(λ_1_ ≥ λ_2_ ≥ 0); λ_1_ and λ_2_ reflect the value of energy change in the direction of its corresponding feature vector. In the consistent region of the image, the intensity value changes a little or almost unchanges, that is, λ_1_ ≈ λ_2_ ≈ 0; in the boundary region, the intensity value across the edge varies greatly, that is, λ_1_ ≥ λ_2_ ≈ 0; the intensity value varies in all directions at the corners, that is, λ_1_ > λ_2_ ≥ 0.

In order to measure the role of each pixel in the image geometry, the following local structure descriptors are extracted from the local structure tensor, the trace C_1_ = λ_1_ + λ_2_ of the local structure tensor is used to describe the strength of the local variation, C_2_ = (λ_1_ − λ_2_)^2^ is defined to characterize the consistency of the local structure, and the scale vector C(x) =[C_1 _C_2_] is defined to represent the structural information adjacent to the pixel, which has better image structure expression ability. Then the weighting function is defined as follows:

(9) $$W\left(x,f_{R},f_{F}\right)=B\left(x\right)\cdot \exp\left(-\left||C\left(x,f_{R}\right)-C\left(x,f_{F}\right)\right|{|}^{2}/\theta \right)$$

where θ is a constant, $B\left(x\right)=1+{\left(\left|\nabla f_{R}\left(x\right)\right|\left|\nabla f_{F}\left(x\right)\right|\right)}^{1/4}$, ∇ *f_R_(x)* and ∇ *f_F_(x)*, respectively, represent gradient vectors of the reference image and the floating image at x pixel points.

The choice of B(x) mainly considers the balance between the strong gradient region and the small gradient region, it has a large value at the strong gradient position and a relatively small value at the small gradient position. The final similarity measure can be expressed as:

(10) $$ALST-NMI\left(x,f_{R},f_{F}\right)=W\left(x,f_{R},f_{F}\right)\cdot NMI\left(x,f_{R},f_{F}\right)$$

## 3 RESULTS AND DISCUSSION

### 3.1 Single‐modality experiment and results

**Figure 2:**
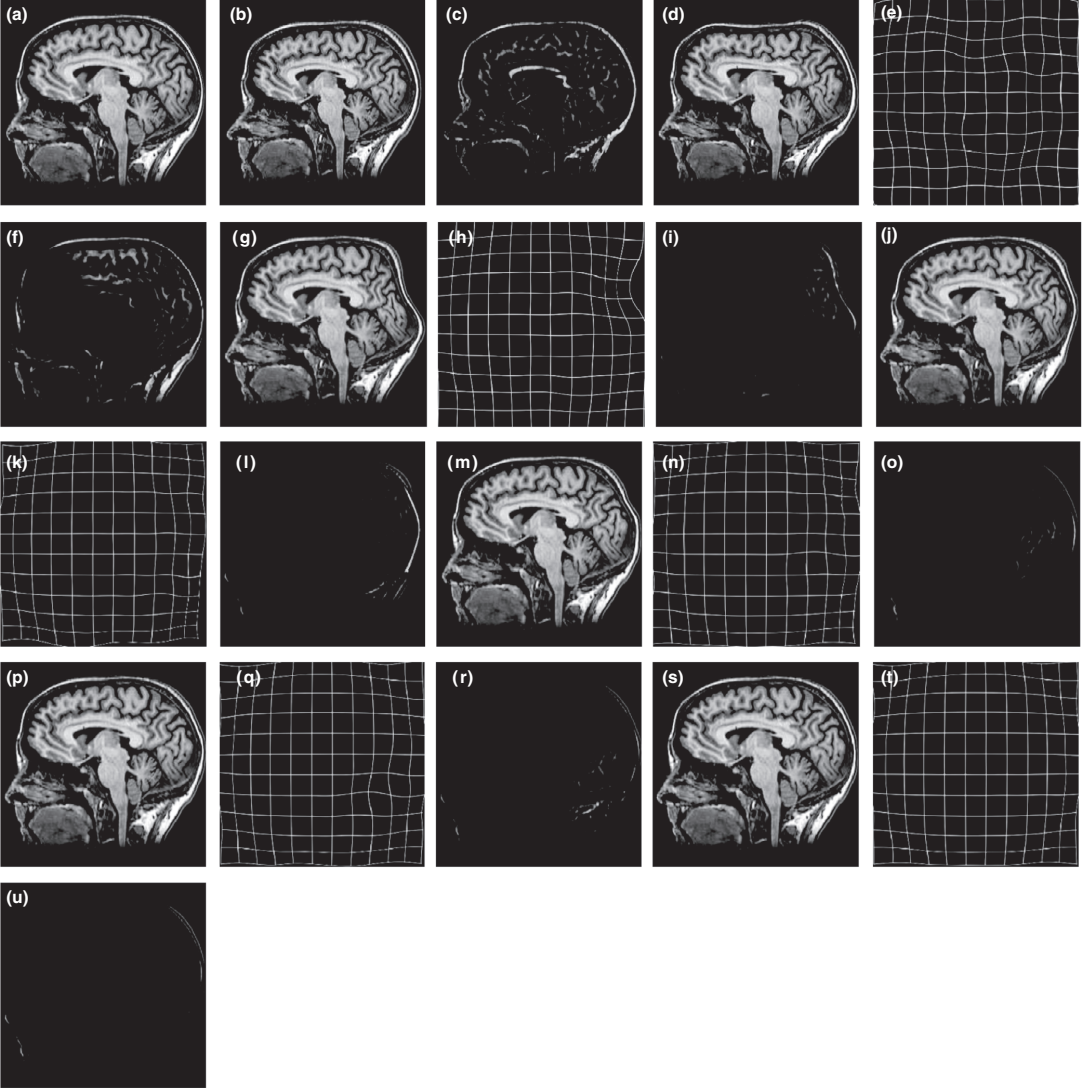
Brain image single‐modality registration result. (a) Reference image; (b) floating image; (c) registration error (a) and (b); (d) gradient difference (GD) deformed image; (e) GD deformation field; (f) registration errors (a) and (d); (g) sum of squared distances (SSD) deformed image; (h) SSD deformation field; (i) registration error (a) and (g); (j) normalized mutual information (NMI) deformed image; (k) NMI deformation field; (l) registration errors (a) and (j); (m) B‐spline and regional mutual information (BRMI) deformed image; (n) BRMI deformation field; (o) registration error (a) and (m); (p) local structure tensor‐normalized mutual information (LST‐NMI) deformed image; (q) LST‐NMI deformation field; (r) registration error (a) and (p); (s) adaptive local structure tensor‐normalized mutual information (ALST‐NMI) deformed image; (t) ALST‐NMI deformation field; (u) registration error (a) and (p).

In order to validate the robustness and accuracy of ALST‐NMI measure, the method is applied for nonrigid medical image registration and compared with SSD, NMI, BRMI and LST‐NMI measure. All the experiments are conducted on a set of real brain images from the brain web with size of 353 × 354. The reference image is shown in Fig. 2(a), the floating image is shown in Fig. 2(b) and 2(b) is obtained by forming the Fig. 2(a) artificially, the grid interval of the transformation model is set as [32 32], and the iterations of the LBFGS optimization algorithm is set as 80. The experimental results are shown in the following figure.

#### 3.1.1 Registration performance evaluation

In order to objectively evaluate the results of registration, this paper uses mean square error, structural similarity index, NMI, and correlation coefficient to quantitatively evaluate the performance of registration.

1. Mean square error

(11) $$MSE=\frac{1}{\mathrm{mn}}\sum_{i=1}^{n}\sum_{j=1}^{m}{\left|R(i,j)-F(i,j)\right|}^{2}$$

where *R(i, j)* and *F(i, j)* represents pixel in the reference image and the floating image, respectively, m × n represent the resolution of the image.

- Structural similarity index

(12) $$SSIM\left(R,F\right)=\frac{\left(2\mu_{R}\mu_{F}+C_{1}\right)\left(2\sigma_{\mathrm{RF}}+C_{2}\right)}{\left(\mu_{R}^{2}+\mu_{F}^{2}+C_{1}\right)\left(\sigma_{R}^{2}+\sigma_{F}^{2}+C_{2}\right)}$$

where μ_R, _μ_F, _σ_R_, σ_F_, and σ_RF_ represents the mean, variance, and covariance of the images R and F, respectively. C_1_ = (k_1_L)^2 ^and C_2_ = (k_2_L)^2 ^are constants used to maintain stability, and L is the dynamic range of the pixel value, k_1_ = 0.01, k_2_ = 0.03.

- Mutual information

(13) MI(R,F)=H(R)+H(F)-H(R,F)

where H(R) and H(F), respectively, denote the information entropy of reference image R and float image F, and H(R,F) is the joint entropy of two images.

- Normalized mutual information

(14) $$NMI\left(R,F\right)=\frac{H(R)+H(F)}{H(R,F)}$$

- Correlation coefficient

(15) $$CC=\frac{\sum_{i=0}^{m}\sum_{j=0}^{n}\left[R\left(i,j\right)-\bar{R(i,j)}\right]\left[F\left(i,j\right)-\bar{F(i,j)}\right]}{\sqrt{\sum_{i=0}^{m}\sum_{j=0}^{n}{\left[R\left(i,j\right)-\bar{R(i,j)}\right]}^{2}}\sqrt{\sum_{i=0}^{m}\sum_{j=0}^{n}{\left[F\left(i,j\right)-\bar{F(i,j)}\right]}^{2}}}$$

where $\bar{R(i,j)}$ and $\bar{F(i,j)}$ represents the mean of pixel in the reference image and the floating image, namely.

- Jacobian determinant

Whether the displacement vector field (DVF) has the ability to keep the image topology unchanged can be measured by Jacobian determinant of the DVF. The value of the Jacobian determinant is larger than zero, indicating that the DVF has the ability to maintain the topology unchanged, the percentage of pixels with negative Jacobian determinant values is expressed as NJ (negative Jacobian). Equation (15) gives the definition of the Jacobian determinant. T = (X, Y) represent the vector of DVF, X and Y respectively represent the position of the point p(x, y) after deformation, then the Jacobian determinant of the DVF at point p is:

(16) $$J_{T}\left(p\right)=\det\left[\partial X/\partial x\;\partial X/\partial y;\partial Y/\partial x\;\partial Y/\partial y\right]$$

- Computation time

All experiments are performed on the MATLAB 2016a, with the Intel Core i7‐4790CPU 3.60GHZ processor and memory is 8G. We provide the values of the computation time under the same computing power for each metrics used.

**Figure 3:**
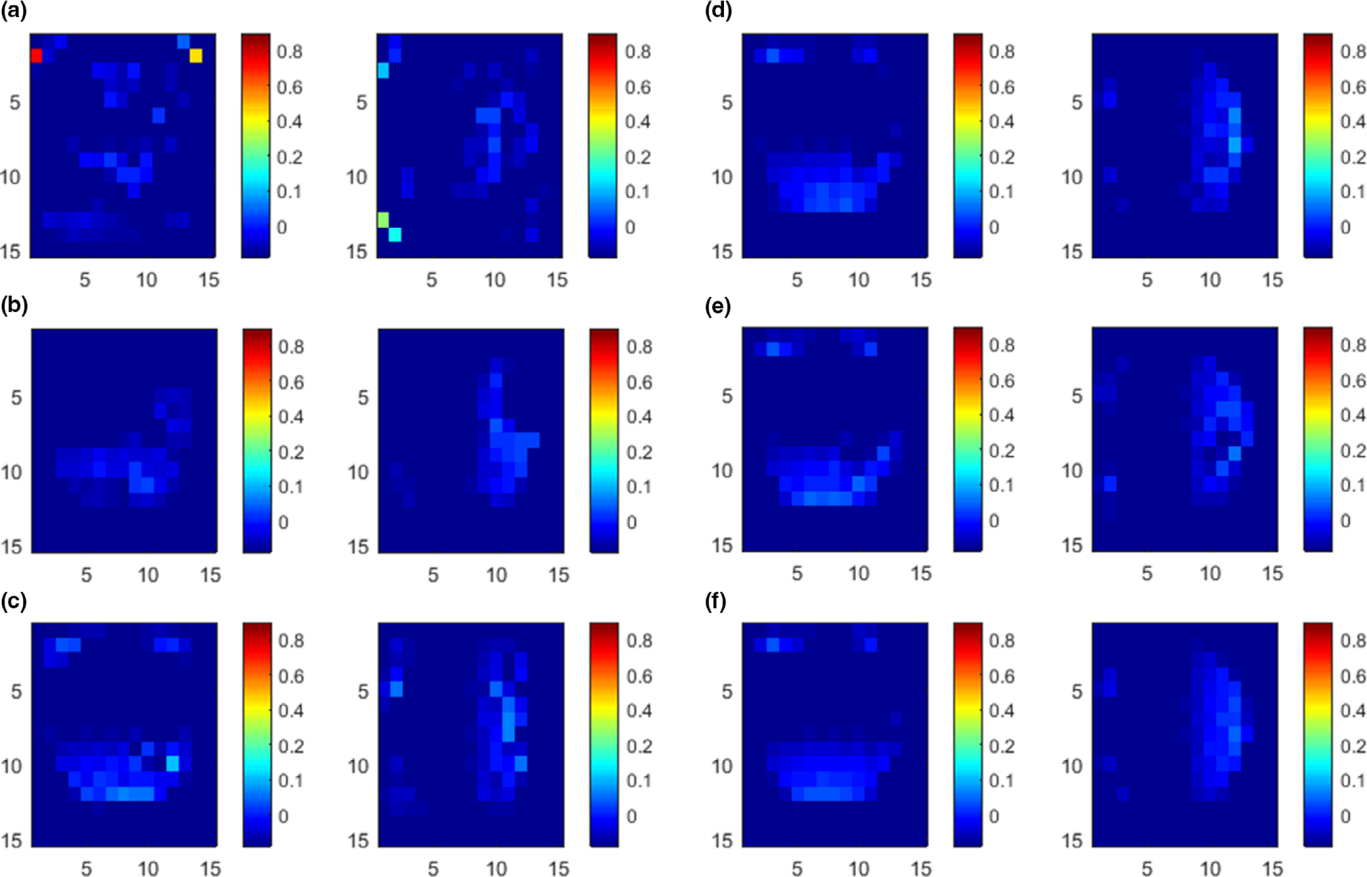
Displacement vector field (DVF) of single‐modality registration. (a) Gradient difference DVFs along Y direction are on the left column, and DVFs along X direction are on the right column. (b) Sum of squared distances; (c) normalized mutual information; (d) B‐spline and regional mutual information; (e) local structure tensor‐normalized mutual information; (f) adaptive local structure tensor‐normalized mutual information.

The results of ALST‐NMI and GD, SSD, NMI, BRMI, LST‐NMI are shown in Fig. 2(d), 2(g), 2(j), 2(m), 2(p), 2(s), 2(e), 2(h), 2(k), 2(n), 2(q), and 2(t) is the transformation image obtained by the corresponding method, and the registration error image shows the distribution of registration errors between the result image and reference image using different methods. It can be seen from Fig. 2(f), 2(i), and 2(l) that, the GD,SSD and NMI measures do not have a good registration result which are calculated by using gradient or intensity information alone, especially perform worse at the edge of the image and the large deformation area of the occipital lobe. It can be seen from Fig. 2(o) and 2(u) that, after combining the spatial information with the ALST‐NMI and BRMI measures, the registration accuracy is significantly improved, and the global contour of the image is basically successfully registered, the large deformation region such as the occipital lobe has also achieved a good result. However, as shown in Fig. 2(m) and 2(s), the registration image obtained by BRMI measure shows a distinct sunken at the edge of the brain, the ALST‐NMI measures can obtain a smooth registration image that is closer to the reference image, and the difference is also minimal compared with the reference image. It can be seen from Fig. 2(r) and 2(u) that, the LST‐NMI measure uses Gaussian filtering with fixed variance and template globally, whether small or large filtering parameters are used, a good registration effect cannot be achieved for the large deformation region of the occipital lobe (Fig. 3). After adding adaptive Gaussian filtering with adaptive variance and template, the large deformation region is successfully registered. Table 1 gives the quantitative metrics of the registration results, it is shown from table that ALST‐NMI measures has improved registration results in all metrics compared to other methods. Compared with the B‐spline + LST‐NMI, the mean square error decreases by 70.17%, the structural similarity increases by 8.09%, the NMI increased by 7.28%, and the correlation coefficient increased by 2.38%, the effectiveness of adding adaptive Gaussian filtering to local structure tensors is demonstrated. Compared with the B‐spline + NMI, the mean square error decreases by 84.87%, the structural similarity increased by 11.6%, the NMI increased by 11.13%, and the correlation coefficient increased by 5.77%, the accuracy of image registration is improved, and the effectiveness of the ALST‐NMI measure is proved.

**Table 1:** Evaluation of single‐modality brain image registration effect

Registration method	MSE	SSIM	NMI	CC	NJ	TIME（S）
Before registration	2732.739	0.361	1.124	0.578	—	—
B‐spline + GD	1808.806	0.569	1.142	0.720	1.78%	35.94
B‐spline + SSD	431.708	0.823	1.221	0.934	0%	2.45
B‐spline + NMI	425.439	0.862	1.312	0.936	0%	517.54
B‐spline + BRMI	131.897	0.894	1.300	0.979	0%	1034.27
B‐spline + LST‐NMI	215.750	0.890	1.359	0.967	0%	738.83
B‐spline + ALST‐NMI	64.350	0.962	1.458	0.990	0%	677.51

MSE, mean‐square error; SSIM, structural similarity index; CC, correlation coefficient; NMI, normalized mutual information; NJ, negative Jacobian; GD, gradient difference; SSD, sum of squared distances; BRMI, B‐spline and regional mutual information; ALST‐NMI, adaptive local structure tensor‐normalized mutual information.

**Figure 4:**
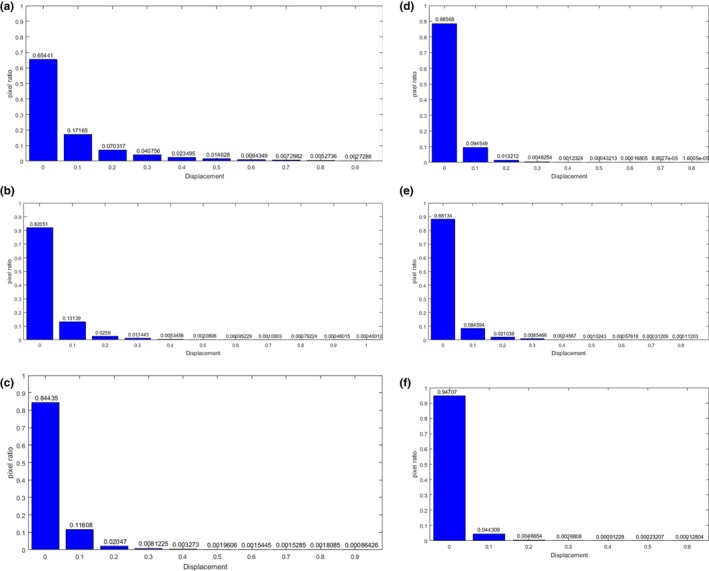
Histogram of the displacement error distribution of the single‐modality registration. (a) gradient difference (b) sum of squared distances; (c) normalized mutual information; (d) B‐spline and regional mutual information; (e) local structure tensor‐normalized mutual information; (f) adaptive local structure tensor‐normalized mutual information.

Figure 4 shows the histogram of the displacement error distribution of the single‐modality registration. Compared with other methods, the registration accuracy of the proposed method is significantly improved, 94.7% of the pixels have been effectively registered, and the percentage of displacement errors of each length in the pixel points has decreased. There is no excessive displacement error, and the maximum error is only 0.6 cm, the error larger than 0.2 cm is less than 0.4% of the total pixels.

### 3.2 Multimodality experiment and results

**Figure 5:**
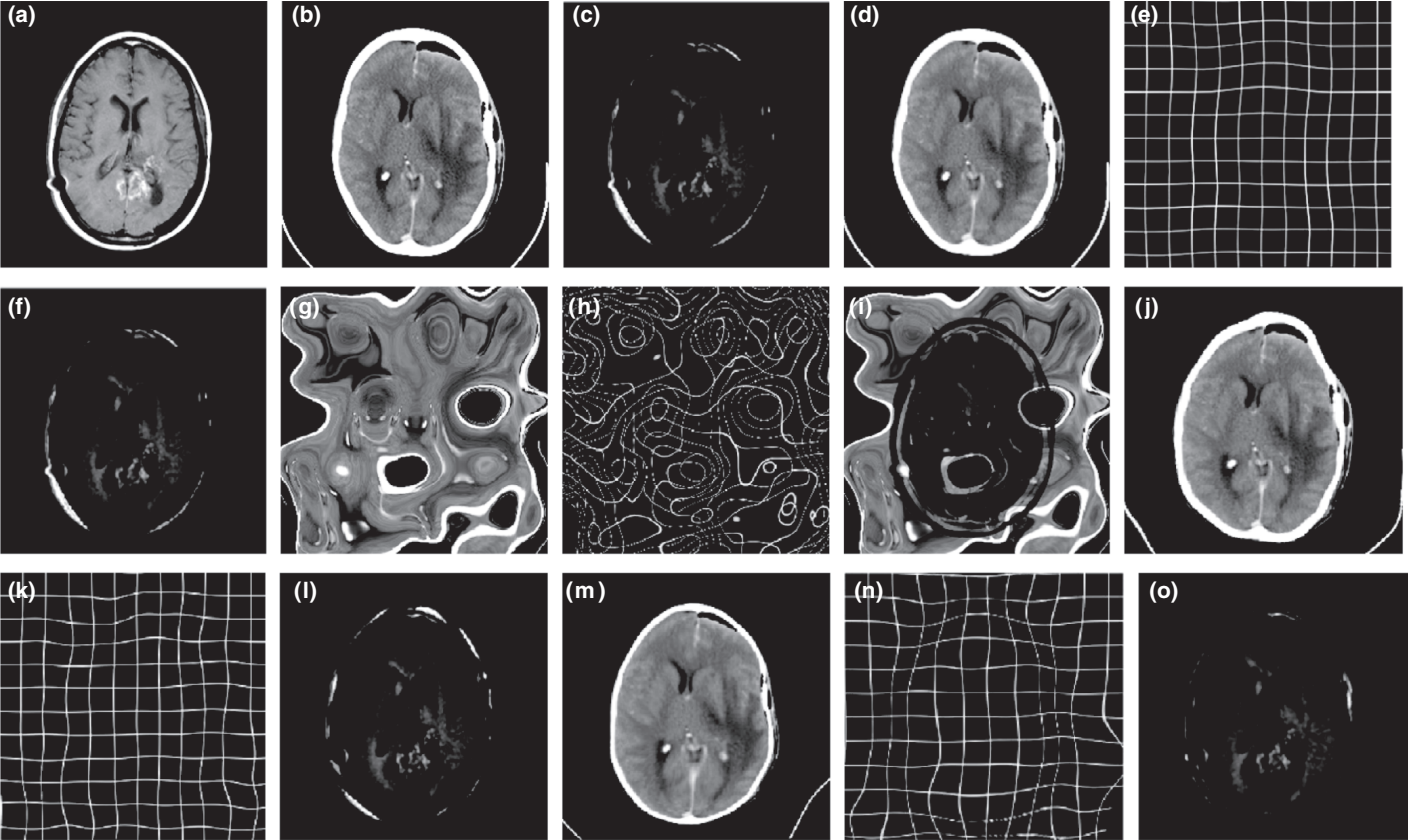
Brain image multimodality registration result. (a) MRI reference image; (b) CT floating image; (c) registration error (a) and (b); (d) gradient difference (GD) deformed image; (e) GD deformation field; (f) registration error (a) and (d); (g) normalized mutual information (NMI) deformed image; (h) NMI deformation field; (i) registration error (a) and (g); (j) local structure tensor‐normalized mutual information (LST‐NMI) deformed image; (k) LST‐NMI deformation field; (l) registration error (a) and (j); (m) adaptive local structure tensor‐normalized mutual information (ALST‐NMI) deformed image; (n) ALST‐NMI deformation field; (o) registration error (a) and (m).

In order to validate the registration results of ALST‐NMI measure on multimodality images, MRI and CT brain images were selected for image registration experiments. The experimental image size is 256 × 256. MRI reference image is shown in Fig. 5(a), MRI floating image is shown in Fig. 5(b), the grid interval of the transformation model is set as [22 22], and iterations of the LBFGS optimization algorithm is set as 30. In the single‐modality medical image registration experiment, the SSD measure was applied for registration and compared with the method proposed in this paper. but SSD measure is not suitable for multi‐modality image registration,so The ALST‐NMI measure is compared with GD, NMI and LST‐NMI measure for multi‐modality image registration, the experimental results are shown in the following figure

**Figure 6:**
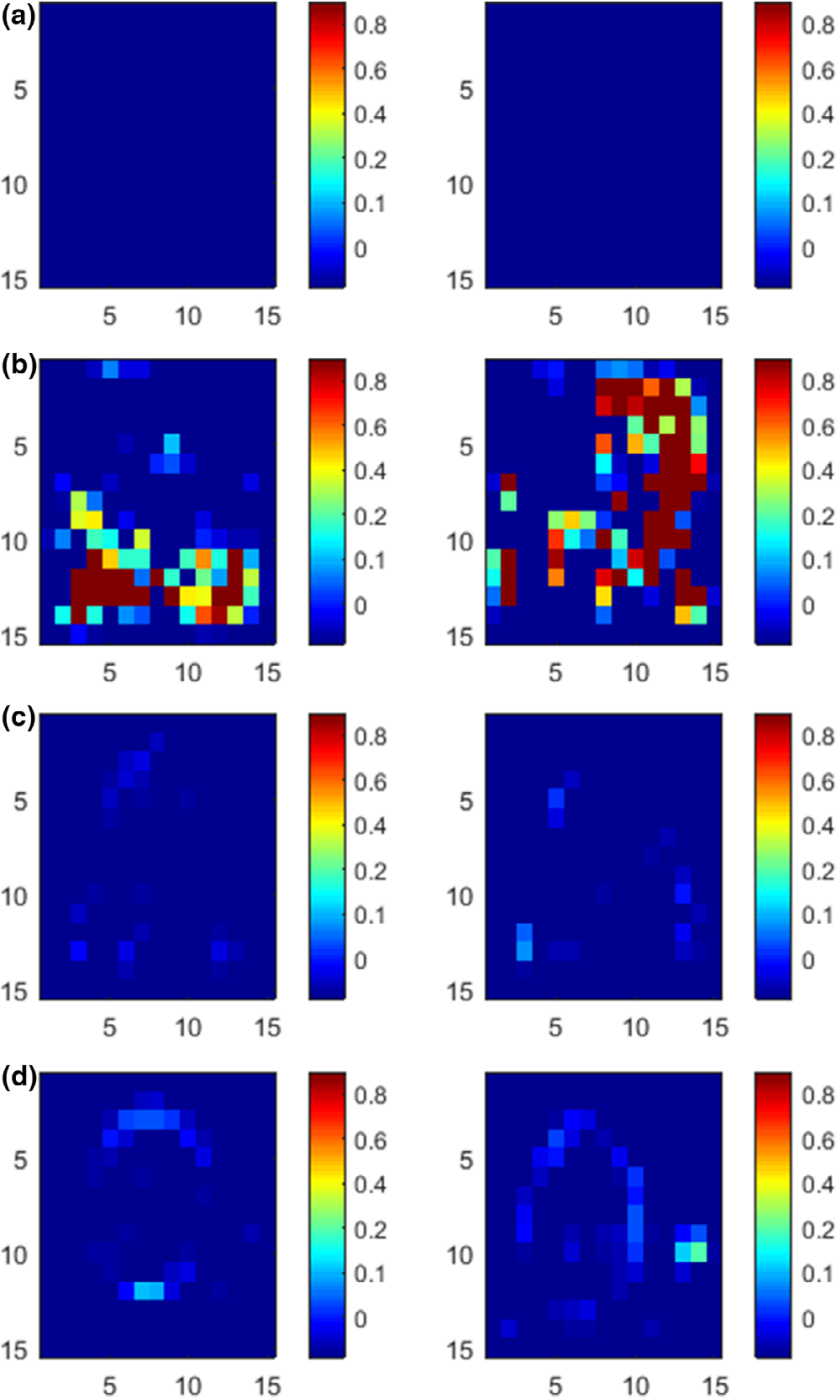
Displacement vector field (DVF) of multimodality registration. (a) gradient difference DVFs along Y direction are on the left column, and DVFs along X direction are on the right column. (b) normalized mutual information (c) local structure tensor‐normalized mutual information; (d) adaptive local structure tensor‐normalized mutual information.

The (d), (g), (j), and (m) of Fig. 5 is the registration result of the ALST‐NMI, GD, NMI, and LST‐NMI measure, the (e), (h), (k), and (n) of Fig. 5 is the transformation image obtained by the corresponding method, and the third column is the registration error image between the registration result image and reference image. It can be seen from Fig. 5(d), 5(j), and 5(m) that the GD, LST‐NMI, and ALST‐NMI measure can successfully register based on the multimodality images, but original nonexistent organization appears on the result image produced by NMI. It can be seen from Fig. 5(h) that the transform mesh grid of the NMI measure appears overlap and the registration fails. The main reason is that multimodality images are obtained by using different imaging principles and devices, the intensity difference of different tissues in different imaging modes is very large, while the NMI measure only uses intensity information for registration, which causes the search optimization algorithm to fall into local extremes and lead to misregistration. Figure 5(m) shows that the ALST‐NMI measure can obtain a smooth image that is closer to the reference image; it reduces the risk of falling into the local extremes and enhances the robustness of the registration compared with the NMI measure. Table. 2 shows the quantitative metrics of various measure registration results. It can be seen that result produced by B‐spline + ALST‐NMI has been improved observably compared with the B‐spline + LST‐NMI, which proves that the combination of local Gaussian filtering and local structure tensor is effective for multimodality images as well. Compared with the B‐spline + LST‐NMI, the mean square error decreases by 35.88%, the structural similarity increases by 12.91%, the NMI increases by 16.15%, and the correlation coefficient increases by 18.64%. The experimental results show that the ALST‐NMI measure can improve the registration accuracy, and can achieve satisfactory results for the nonrigid registration of large deformation multimodality medical images (Fig. 6).

**Table 2:** Evaluation of multimodality brain image registration effect

Registration method	MSE	SSIM	MI	CC	NJ	TIME(S)
Before registration	4806.17	0.536	0.567	0.554	—	—
B‐spline + GD	4203.41	0.55	0.644	0.601	0%	16.65
B‐spline + NMI	10059.58	0.109	0.159	0.041	33.78%	170.14
B‐spline + LST‐NMI	3497.85	0.580	0.682	0.657	0%	188.91
B‐spline + ALST‐NMI	2695.14	0.621	0.748	0.713	0%	217.14

**Figure 7:**
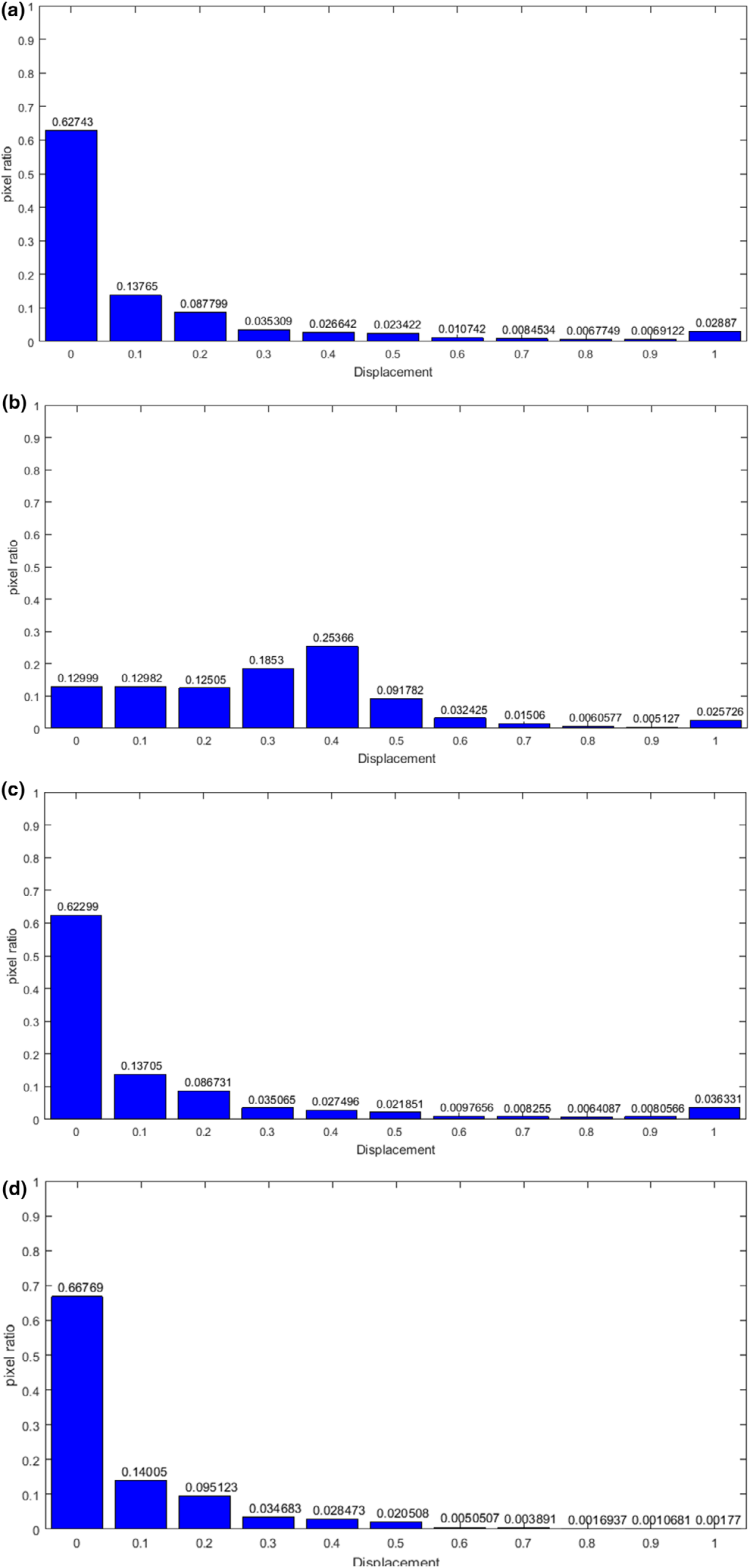
Histogram of the displacement error distribution of the multimodality registration. (a) gradient difference (b) normalized mutual information (c) local structure tensor‐normalized mutual information; (d) adaptive local structure tensor‐normalized mutual information.

Figure 7 shows the histogram of the displacement error distribution of the multimodality registration. Compared with the GD method, the ALST‐NMI measure has obvious improvement for large displacement error, the pixels with error of 1 cm decreases from 2.88% to 0.22%. Compared with the LST‐NMI method, the mean error is significantly reduced, which proves the validity of adaptive local structure tensor. 66.75% of the pixels have been effectively registered, and the error larger than 0.5 cm is less than 2% of the total pixels.

## 4 CONCLUSION

In this paper, a similarity measure based on adaptive local structure tensor and NMI is proposed for nonrigid medical image registration with large deformation, in which intensity information and spatial information are both considered. ALST‐NMI similarity measure uses a weighting function to balance the registration of strong gradient regions and small gradient regions, the experiments show that the B‐spline + ALST‐NMI can effectively improve the image registration accuracy. However, since the NMI and the local structure tensor of the discrete points need to traverse each pixel, the registration efficiency is reduced to a certain extent. It is necessary that how to achieve higher registration precision and computational efficiency simultaneously.

## CONFLICT OF INTEREST

The authors declare no conflict of interest.

## References

[1] Gottesfeld BL . A survey of image registration techniques. ACM Comput Surv. 1992;24:325–376.

[2] Burt PJ , Yen C , Xu X . Local correlation measures for motion analysis. Proceedings of IEEE Pattern Recognition and Image Processing Conference. 1982;269–274.

[3] Klein S , Staring M , Murphy K , Viergever Ma , Pluim J . Elastix: a toolbox for intensity‐based medical image registration. IEEE Trans Med Imaging. 2009;29:196–205. 19923044 10.1109/TMI.2009.2035616

[4] Viola P , Wells WM . Alignment by maximization of mutual information. Proceedings of IEEE International Conference on Computer Vision. 2002.

[5] Studholme C , Hill DLG , Hawkes DJ . An overlap invariant entropy measure of 3D medical image alignment. Pattern Recogn. 1999;32:71–86.

[6] Pluim JPW , Maintz J , Viergever MA . Image registration by maximization of combined mutual information and gradient information. IEEE Trans Med Imaging. 2002;19:809–814. 10.1109/42.876307 11055805

[7] Russakoff DB , Tomasi C , Rohlfing T , Maurer CR . Image similarity using mutual information of regions In European Conference on Computer Vision‐eccv. Berlin, Heidelberg: Springer; 2004:596–607.

[8] Loeckx D , Slagmolen P , Maes F , Vandermeulen D , Suetens P . Non‐rigid image registration using conditional mutual information. IEEE Trans Med Imaging. 2010;29:725–737. 10.1109/TMI.2009.2021843 19447700

[9] Rivaz H , Karimaghaloo Z , Collins DL . Self‐similarity weighted mutual information: A new nonrigid image registration metric. Med Image Anal. 2014;18:343–358. 24412710 10.1016/j.media.2013.12.003

[10] Luan H , Qi F , Xue Z , Chen L , Shen D . Multimodality image registration by maximization of quantitative‐qualitative measure of mutual information. Pattern Recogn. 2008;41:285–298.

[11] Hossny M , Nahayandi S , Creighton D , Bhatti A . Image fusion performance metric based on mutual information and entropy driven quadtree decomposition. Electron Lett. 2010;46:1266–1268.

[12] Jun W , Fengmei L . Nonrigid medical image registration based on P‐spline and regional mutual information. Appl Res Comput. 2017;34:1001–3695.

[13] Qu J , Lei J , Li Y , Dong W , Zeng Z , Chen D . Structure tensor‐based algorithm for hyperspectral and panchromatic images fusion. Remote Sens. 2018;10:373.

[14] Sloan JM , Goatman K , Siebert P . FASTR: using local structure tensors as a similarity metric. Procedia Comput Sci. 2016;90:194–199.

[15] Zhiyong H , Xin C , Lining S . Saliency mapping enhanced by structure tensor. Comput Intell Neurosci. 2015; 2015: 1–8. 10.1155/2015/875735 PMC4691604 26788050

[16] Surya Prasath VB , Vorotnikov D , Pelapur R , Jose S , Seetharaman G , Palaniappan K . Multiscale Tikhonov‐total variation image restoration using spatially varying edge coherence exponent. IEEE Trans Image Process. 2015; 24: 5220–5235. 26394419 10.1109/TIP.2015.2479471

[17] Estellers V , Soatto S , Bresson X . Adaptive regularization with the structure tensor. IEEE Trans Image Process. 2015; 24: 1777–1790. 25769155 10.1109/TIP.2015.2409562

[18] Fan R , Wenlong L , Xuming Z , Zhouping Y . Non‐rigid registration of multi‐modality medical image using combined gradient information and mutual information. J Med Imaging Health Inform. 2018; 8: 1374–1383.

